# Application of updated guidelines on diastolic dysfunction in patients with severe sepsis and septic shock

**DOI:** 10.1186/s13613-017-0342-x

**Published:** 2017-12-19

**Authors:** David J. Clancy, Timothy Scully, Michel Slama, Stephen Huang, Anthony S. McLean, Sam R. Orde

**Affiliations:** 1 ICU, Nepean Hospital, Kingswood, NSW 2747 Australia; 2 Medical ICU, Amiens University Hospital, Amiens, France

**Keywords:** Sepsis, Diastolic function, Systolic function

## Abstract

**Background:**

Left ventricular diastolic dysfunction is suggested to be associated with higher mortality in severe sepsis and septic shock, yet the methods of diagnosis described in the literature are often inconsistent. The recently published 2016 American Society of Echocardiography and European Association of Cardiovascular Imaging (ASE/EACVI) guidelines offer the opportunity to apply a simple pragmatic diagnostic algorithm for the detection of diastolic dysfunction; however, it has not been tested in this cohort.

**Aims:**

We sought to assess the applicability in septic patients of recently published 2016 ASE/EACVI guidelines on diastolic dysfunction compared with the 2009 ASE guidelines. Our hypothesis was that there would be poor agreement in classifying patients.

**Methods:**

Prospective observational study includes patients identified as having severe sepsis and septic shock. Patients underwent transthoracic echocardiography on day 1 and day 3 of their ICU admission. Patients with normal and abnormal (ejection fraction < 52%) systolic function had their diastolic function stratified according to both the 2009 ASE and 2016 ASE/EACVI guidelines.

**Results:**

On day 1 echocardiography, of the 62 patients analysed, 37 (60%) had diastolic dysfunction according to the 2016 ASE/EACVI guideline with a further 23% having indeterminate diastolic function, compared to the 2009 ASE guidelines where only 13 (21%) had confirmed diastolic dysfunction with 46 (74%) having indeterminate diastolic dysfunction. On day 3, of the 55 patients studied, 22 patients (40%) were defined as having diastolic dysfunction, with 6 (11%) having indeterminate diastolic dysfunction according to the 2016 ASE/EACVI guidelines, compared to the 2009 guidelines where 11 (20%) were confirmed to have diastolic dysfunction and 41 (75%) had indeterminate diastolic function. Systolic dysfunction was identified in 18 of 62 patients (29%) on day 1 and 18 of 55 (33%) on day 3. These patients were classified as having abnormal diastolic function in 94 and 89% with the 2016 guidelines on day 1 and day 3, respectively, compared with 50 and 28% using the 2009 guidelines. The 2016 guidelines had less patients with indeterminate diastolic function on days 1 and 3 (11 and 6%) compared to the 2009 guidelines (50 and 72%). Normal systolic function was identified in 44 patients on day 1 and 37 on day 3. In this group, abnormal diastolic function was present in 45 and 54% on days 1 and 3 according to the 2016 ASE/EACVI guidelines, compared with 9 and 16% using the 2009 guidelines, respectively. In those with normal systolic function, the 2016 guidelines had less indeterminate patients with 30 and 16% on days 1 and 3, respectively, compared to 84 and 76% in the 2009 guidelines.

**Conclusion:**

The 2016 ASE/EACVI diastolic function guidelines identify a significantly higher incidence of dysfunction in patients with severe sepsis and septic shock compared to the previous 2009 guidelines. Although the new guidelines seem to be an improvement, issues remain with the application of guidelines using traditional measures of diastolic dysfunction in this cohort.

**Electronic supplementary material:**

The online version of this article (10.1186/s13613-017-0342-x) contains supplementary material, which is available to authorized users.

## Background

Systolic and diastolic dysfunction occur frequently in severe sepsis and septic shock [1]. Whilst systolic dysfunction has been suggested not to be associated with mortality [2], there is conflicting evidence in regard to diastolic dysfunction and its effect on mortality in sepsis [3–12]. One of the major issues in research in this field to date is the large variation in diagnostic criteria used to define diastolic dysfunction [7, 8, 11, 13], which limits the interpretation of subsequent analyses [4]. Previous guidelines from the American Society of Echocardiography (ASE) [14] have been limited by several factors, for example the mandatory inclusion of left atrial size that is assumed to increase in response to raised left atrial pressures [13, 15]. This may not be the case in the acute situation. The most recent recommendations from the ASE and the European Association of Cardiovascular Imaging (ASE/EACVI) published in 2016 [16] have significant advantages, including increased flexibility, with recognition that not all parameters (i.e. left atrial size) are abnormal in diastolic dysfunction. Furthermore, they recognize that given the relationship between systolic function and myocardial relaxation that patients with abnormal systolic function or structural abnormalities must automatically have a degree of impaired diastolic function. Hence, they have prescribed an approach whereby those with normal systolic function need to have impairment of diastolic function detected before subsequent grading of severity, whereas those with abnormal systolic function or structural issues must have impaired relaxation and subsequently can proceed to grading of their diastolic dysfunction. The parameters used in the algorithms have been simplified, with less importance placed on parameters that are difficult to measure in the intensive care unit. The authors of these guidelines note that they are applicable to the general population seen in an ambulant setting, but not in children or in the peri-operative setting. In the absence of an accepted gold standard for diastolic dysfunction, these same guidelines are utilized to make the diagnosis of diastolic dysfunction in the critically ill population. However, despite improvements made in defining diastolic dysfunction, caveats remain with each parameter that can make the recognition of impaired relaxation difficult in the critically ill patients.

We sought to compare the 2009 ASE and the recent 2016 ASE/EACVI algorithms for diagnosing diastolic dysfunction in a population with severe sepsis and septic shock to assess and compare their ability to detect and differentiate grades of diastolic dysfunction. Our hypothesis was that there would be poor agreement in classifying patients with diastolic dysfunction between the 2009 and 2016 guidelines for diastolic dysfunction.

## Methods

We conducted a prospective, observational cohort study at the Nepean Hospital Intensive Care Unit, Sydney, NSW, from September 2014 to February 2016. The study was approved by the Nepean Blue Mountains Local Health District Research Governance Office (14/35-LR/14/Nepean/70). As echocardiography is a standard procedure in critically ill patients in our unit, consent was waived. Inclusion criteria were: adult patients (> 18 years) admitted to Nepean ICU with severe sepsis or septic shock based on the previous 2012 Surviving Sepsis guidelines that were current at the time of data collection. Severe sepsis was defined as having a documented or strong suspicion of infection, with at least 2 of 4 clinical signs of inflammation (temperature > 38 or < 36 °C, heart rate > 90 bpm, white blood cell count < 4 or > 12 × 10^9^/L, respiratory rate > 20/min or PaCO_2_ < 32 mmHg) with additional evidence of organ dysfunction. Septic shock is defined as sepsis with refractory hypotension requiring vasoactive treatment [17]. The authors recognize that since completion of enrolment in this study the definition of sepsis and septic shock has changed [18]. Exclusion criteria included: pregnancy, congenital heart disease, artificial valve prosthesis, severe mitral pathology and inadequate image quality.

Patient data collected included: demographic and physiological data, SOFA scores, fluid balances, inotropic use and mechanical ventilation parameters. Previous echo reports (including diastolic dysfunction) when available were acquired, although the grading of diastolic dysfunction for these studies was not based on the 2016 ASE/EACI guidelines. SOFA scores were retrospectively calculated at the time of the echo studies. Current rates of noradrenaline infusion and total volume of noradrenaline infused were also recorded to the nearest hour.

### Echocardiography

A baseline, comprehensive echocardiogram was performed by sonographers or S.O. (intensive care and echocardiography specialist) at the earliest opportunity following admission (day 1). Parameters measured were in accordance with current practice and included: LV size, LV ejection fraction, left atrial volume, mitral inflow velocity, septal and lateral annulus tissue Doppler, tricuspid regurgitation (TR) velocity and cardiac output. Ejection fraction and left atrial volume were measured with Simpson’s biplane technique. Measurements were averaged from 3 cardiac cycles if the patient was in sinus rhythm and 5 cardiac cycles in those with atrial fibrillation. Tissue Doppler measurements were taken from the modal velocity (or peak intensity of the Doppler signal) rather than the peak of the waves, given the variable accuracy of peak tissue Doppler measurements in various machines [19]. A repeat study was performed as soon as feasible from day 3 of admission.

**Fig. 1:**
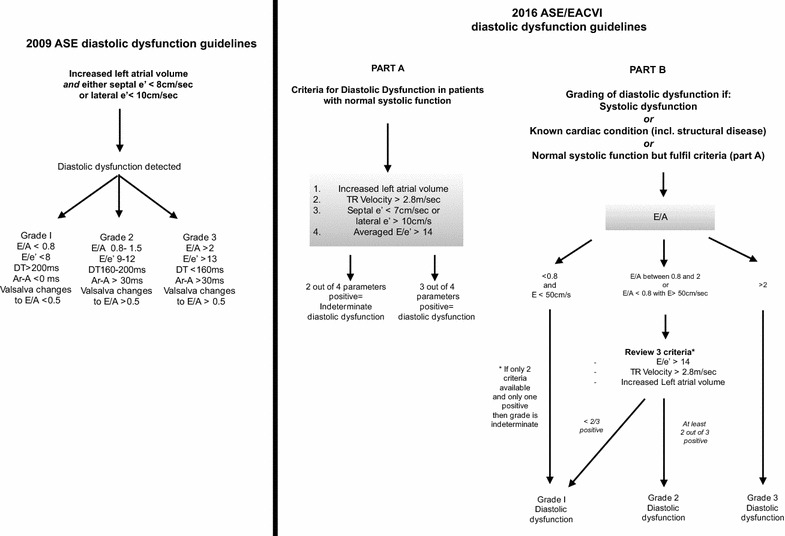
2009 ASE and 2016 ASE/EACVI algorithms for diagnosis of diastolic dysfunction

Systolic dysfunction was defined as those with a calculated ejection fraction (EF) (using Simpson’s biplane method) < 52%. Diastolic dysfunction was classified according to both the 2009 ASE and 2016 ASE/EACVI guidelines (see Fig. 1 for summary of diagnostic algorithms). In regard to the 2009 ASE guidelines, deceleration time was excluded given its limitation in fusion of the *E* and *A* waves due to tachycardia and Valsalva manoeuvres were not performed due to difficulty in performing in the critically ill. Pulmonary venous Doppler parameters were sought if available to aid in the diagnosis, but only to be used if the sample was adequate. The *E*:*e*′ was calculated based on an average of the medial and lateral *e*′ values. Left atrial volume was indexed for body surface area and considered increased if > 34 ml/m^2^. If height and weight data were not available, left atrial volume was considered increased if > 52 ml (for females) and > 58 ml (for males). For the 2016 guidelines, if patients had normal systolic function and no obvious structural abnormalities, they were first screened for diastolic dysfunction via a separate algorithm before subsequent grading, requiring at least 3 of the 4 prescribed parameters to be positive. Those with structural abnormalities, known ischaemic heart disease or abnormal systolic function, given that they will have impaired myocardial relaxation, proceeded directly to grading of diastolic dysfunction.

### Data and statistical analysis

Statistical analysis was performed with the software program JMP version 11 (SAS, Cary, North Carolina, USA). Cohen’s kappa analysis was performed to assess the level of agreement of the 2009 and 2016 guidelines, with the null hypothesis accepted if kappa was greater than 0.7 (considered reasonable agreement) and rejected if kappa was less than 0.4 (considered poor agreement). Using a significance level of 0.05, a sample size of 44 was required to give 90% power of detecting a true difference [20]. Given the risk of missing data or insufficient image quality, this sample size was extended to at least 60 patients. Continuous variables are reported as mean ± standard deviation (SD) or median ± interquartile range (IQR) and are analysed between groups using analysis of variance, and if a significant difference found, individual group analysis was performed by Tukey’s HSD test. Categorical variables are expressed as number of patients and percentage of group, with comparisons made by Pearson’s Chi-squared test or Fisher’s exact test if less than 5 patients were in a specific group. For unadjusted comparisons between groups, a Student’s *t* test was used for normally distributed data and a Wilcoxon signed-rank test for non-normally distributed data. Probability values are considered two-sided, and a *p* value < 0.05 was considered significant. All echocardiograms were reviewed by two different examiners (T.S and D.C) who were blinded to each other’s findings. Measurements taken were in keeping with recommendations from the ASE [16, 21] and as such are reproducible. Grading of diastolic dysfunction was performed by two examiners (M.S and D.C). Any discrepancies were resolved by consensus in the presence of an adjudicator (S.O).

## Results

**Fig. 2:**
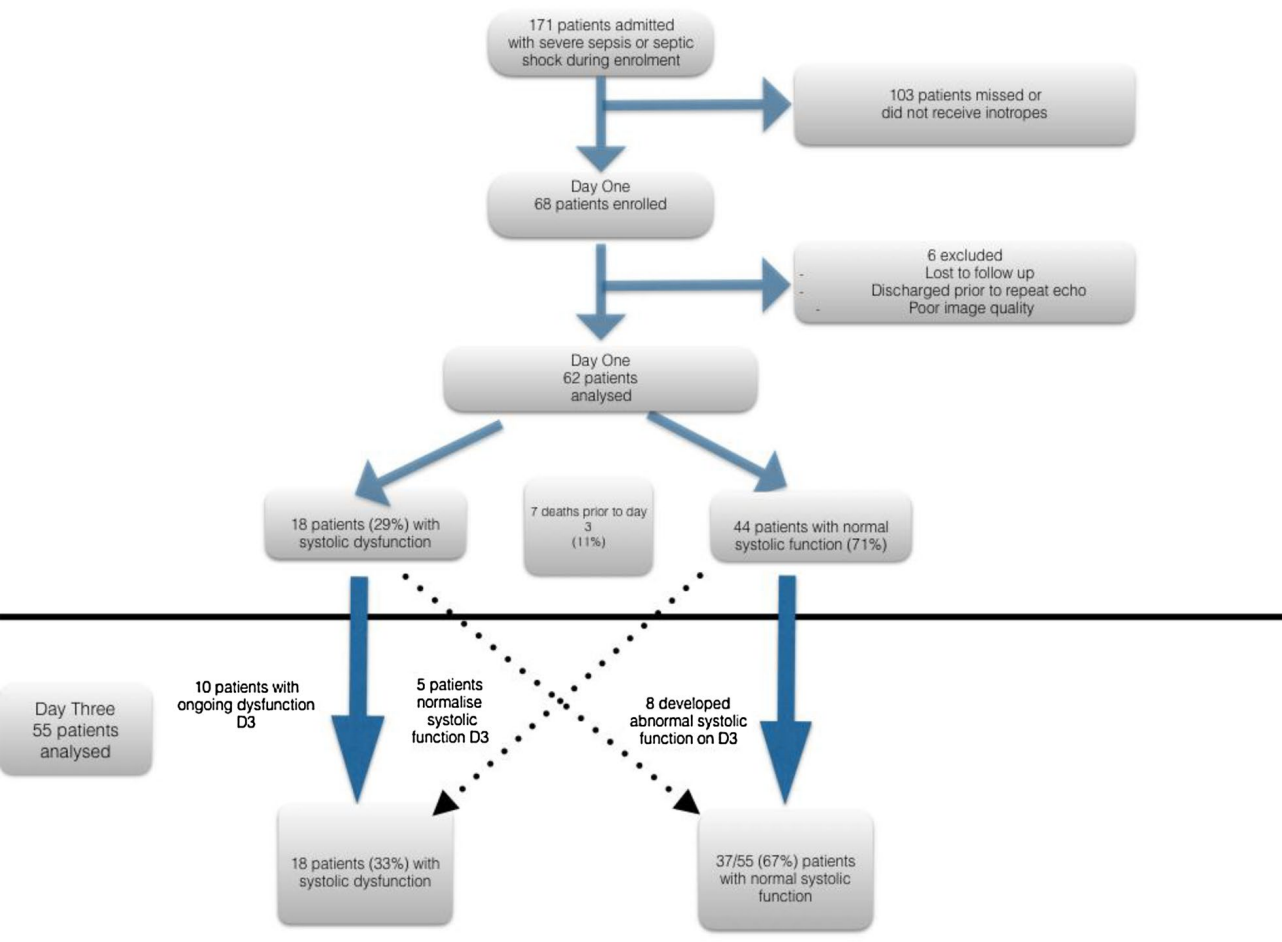
Consort diagram of participants in study

Sixty-eight patients were included in the study (see Fig. 2). Six were lost to follow-up or had insufficient imaging and were excluded from analysis. A further seven patients (11%) died before repeat echocardiography. In total, 15 (24%) patients died in the ICU, with a total of 20 (32%) dying in hospital. Baseline demographics of all patients are included (Table 1). The median time to first echocardiograph was 19 h from admission (IQR 11.5, 31.5) and 90 h (IQR 68, 108) for the repeat echocardiograph.

**Table 1:** Baseline demographics of all patients and those with normal systolic function on day 1

Variable	All patients (62)	Normal ejection fraction on day 1 *N* = 44 (71%)		
		Normal diastolic function (*n* = 11)	Abnormal diastolic dysfunction (*n* = 20)	Indeterminate diastolic function (*n* = 13)
Demographics				
Age	63.1 ± 12.4	55 ± 14	67 ± 8*	62 ± 12
Sex (M)	35 (56%)	6 (55%)	6 (30%)	6 (46%)
Past medical history				
IHD	19 (31%)	0	3 (15%)	5 (38%)
Diabetes	20 (32%)	3 (27%)	5 (25%)	4 (31%)
HTN	37 (60%)	4 (36%)	6 (30%)	6 (46%)
Previous documented diastolic dysfunction	6 (10%)	0	3 (15%)	2 (15%)
Previous documented systolic dysfunction	5 (8%)	1 (9%)	0	0
CRF	11 (18%)	0	5 (25%)	3 (23%)**
Clinical data				
Mechanical ventilation D1 (n)	44 (71%)	7 (64%)	13 (65%)	9 (69%)
Mechanical ventilation D3	32 (52%)	6 (55%)	8 (40%)	9 (69%)
Total noradrenaline at first echo (ml)	155 (51, 330)	49 (15, 162)	129 (47, 330)	202 (111,573)
HR day 1	97 ± 21	97 ± 25	91 ± 17	101 ± 14
Arrhythmia D1	13 (21%)	2 (18%)	4 (20%)	2 (15%)
SOFA D1	10 ± 3.7	9.5 ± 4	9.9 ± 3.9	9.1 ± 3.7
PEEP D1	8 (5, 10)	10 (5, 14)	8 (6, 10)	8 (5,9)

Diastolic dysfunction assessed by 2016 ASE/EACVI guidelines

*p* values not given unless significant, *p < 0.018; **p < 0.021

On day 1 echocardiography, of the 62 patients analysed 37 (60%) had diastolic dysfunction according to the 2016 ASE/EACVI guideline with a further 23% having indeterminate diastolic function, compared to the 2009 ASE guidelines where only 13 (21%) had confirmed diastolic dysfunction with 46 (74%) having indeterminate diastolic dysfunction. The degree of agreement between the two guidelines on day 1 was poor, with Kappa being 0.24 (*p* = 0.0002). On day 3, of the 55 patients studied, 22 patients (40%) were defined as having diastolic dysfunction, with 6 (11%) having indeterminate diastolic dysfunction according to the 2016 ASE/EACVI guidelines, compared to the 2009 guidelines where 11 (20%) were diagnosed to have diastolic dysfunction and 41 (75%) had indeterminate diastolic function. Again agreement was poor (Kappa 0.13, *p* = 0.03). The details of the abnormal parameters with respect to the 2009 and 2016 grading of diastolic dysfunction for days 1 and 3 are included (see Additional files 1 and 2).

**Fig. 3:**
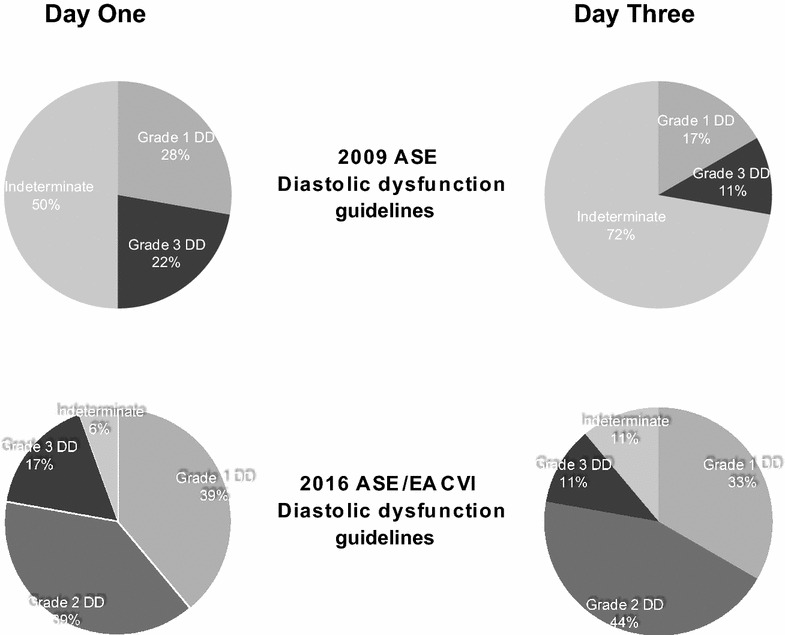
Grading of diastolic dysfunction according to 2009 ASE and 2016 ASE/EACVI algorithms for diagnosis of diastolic function in patients with abnormal systolic function on day 1 and day 3

Systolic dysfunction was identified in 18 patients (29%) on day 1 and 18 of 55 (33%) on day 3 (see Additional file 3). These patients were able to be classified as having abnormal diastolic function in 94 and 89% on day 1 and day 3 with the 2016 guidelines, compared with 50 and 28% with the 2009 guidelines, with the remainder being indeterminate (see Fig. 3). This demonstrates poor agreement between the two guidelines with the kappa coefficient on day 1 of 0.26 (*p* = 0.004) and 0.07 on day 3 (*p* = 0.34).

**Fig. 4:**
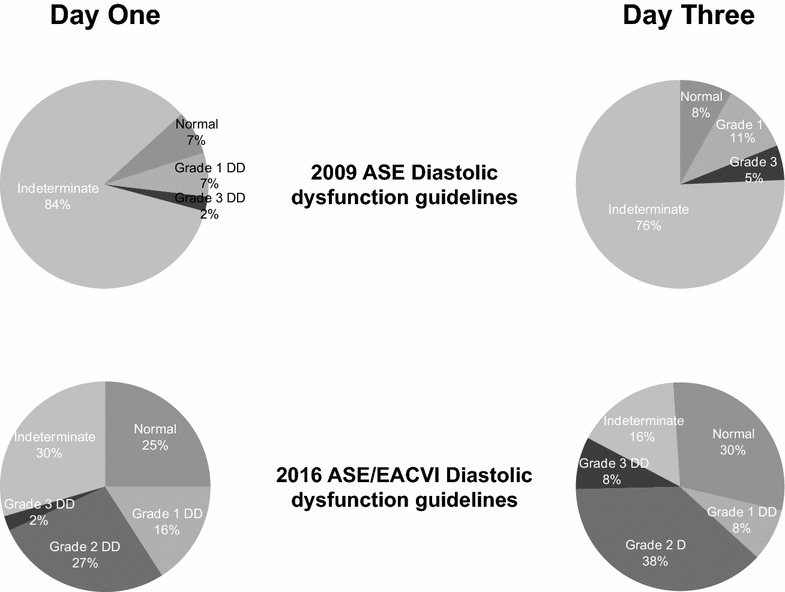
Grading of diastolic dysfunction according to 2009 ASE and 2016 ASE/EACVI algorithms for diagnosis of diastolic function in patients with normal systolic function on days 1 and 3

There were 44 patients on day 1 with a normal ejection fraction. Using the 2016 ASE/EACVI guidelines, 11 (25%) patients had normal diastolic function, with 20 (45%) having diastolic dysfunction and 13 (30%) unable to be determined, whereas using the 2009 guidelines 7% were normal, 9% had diastolic dysfunction and 84% were indeterminate (see Fig. 4). There was poor agreement between the 2009 and 2016 diastolic dysfunction guidelines (kappa coefficient 0.18, *p* = 0.004).

Of those with normal diastolic function on day 1 according to the 2016 guidelines, 3 proceeded to diastolic dysfunction on day 3, with another 2 having indeterminate diastolic function. Seven of the indeterminate patients on day 1 progressed to definite diastolic dysfunction, with 5 having evidence of raised left atrial pressure (grade 2 or 3) on day 3.

On day 3, out of the 37 patients with normal systolic function, 11 (30%) had normal diastolic function, 20 (54%) had diastolic dysfunction and 6 (16%) were indeterminate according to the 2016 guidelines, compared to 8% normal, 16% with diastolic dysfunction and 76% indeterminate using the 2009 guidelines (Fig. 4). Again, this demonstrated poor agreement between the two guidelines (kappa coefficient 0.13, *p* = 0.005). Those with normal systolic function but abnormal diastolic dysfunction tended to be older compared with patients with normal diastolic function on both days. There was no significant difference in noradrenaline requirements, heart rates, SOFA scores, PEEP, or mechanical ventilation on either day 1 (see Table 1) or day 3. Echocardiography parameters for patients with normal systolic function on day 1 are included in Table 2.

**Table 2:** Patients with normal systolic function and their echocardiographic parameters on day 1

Echo parameter	Normal diastolic function *n* = 11 (25%)	Grade I diastolic dysfunction *n* = 7 (16%)	Grade 2 diastolic dysfunction *n* = 12 (27%)	Grade 3 diastolic dysfunction (*n* = 1) (2%)	Indeterminate diastolic function (*n* = 13) (30%)
Septal hypertrophy	0	7 (100%)	5 (42%)	0	4 (31%)
*E*/*e*′ > 14	0	0	8 (67%)	1 (100%)	3 (23%)
Mean *E*/*e*′	9.5 ± 2.4	10 ± 2.3	15.6 ± 4.6	22.4	13 ± 4.9
Septal *e*′ < 7 cm/s (*n*)	6 (55%)	5 (71%)	10 (83%)	1	10 (77%)
Septal *e*′ (cm/s)	0.08 ± 0.02	0.06 ± 0.02	0.055 ± 0.01	0.03	0.055 ± 0.01
Lateral *e*′ < 10 cm/s (*n*)	5 (45%)	4 (57%)	11 (92%)	1	10 (77%)
Mean lateral *e*′ (cm/s)	0.09 ± 0.03	0.08 ± 0.03	0.08 ± 0.02	0.07	0.085 ± 0.02
Increased left atrial volume (*n*)	3 (27%)	3 (43%)	11 (92%)	1	8 (67%)
Mean left atrial volume (ml)	51 ± 12	52 ± 20	87.5 ± 29	111	56 ± 15
TR velocity > 2.8 (*n*)	1 (9%)	0 (0%)	9 (75%)	1	0
TR velocity average (m/s)	2.43 (1.97,2.67)	2.4 (1.6, 2.74)	2.96 (2.8, 3.22)	3.29	2.35 (2.2, 2.52)
Cardiac output (L/min)	5.5 ± 1.5	6.9 ± 2.6	6.2 ± 1.65	N/A	6.05 ± 1.2

Diastolic dysfunction assessed by 2016 ASE/EACVI guidelines

## Discussion

Our results demonstrate an increased detection of diastolic dysfunction in patients with severe sepsis and septic shock using more recent 2016 ASE/EACVI guidelines as compared with the 2009 ASE version. In the absence of a gold standard for diastolic dysfunction, it is unknown whether this is a true reflection of the patient’s diastolic function. However, given the 2009 ASE guidelines have a significant higher percentage of patients with indeterminate diastolic dysfunction compared to the 2016 guidelines, the new guidelines appear to have an improved clinical applicability and should form the reference standard for use in this cohort and in further research in this field. The limitations of the 2009 guidelines are supported by prior studies [13] [7].

There are several advantages in the current guidelines that increase their ability to be applied to patients with severe sepsis and septic shock. Firstly, the recognition that those with systolic dysfunction must have impaired relaxation is an important distinction backed up with long-standing evidence [22]. Previous research regarding diastolic dysfunction in severe sepsis and septic shock has not made this important distinction. Secondly, there is increased flexibility in recognizing that not all parameters may be present in any one patient, which is particularly important when applying the criteria to acute situations. Finally, we note that all of the parameters in this guideline are relatively easy to measure if the clinician is aware of the pitfalls and maintains due diligence with measurements as part of the rigour required for accurately assessing diastolic function. Subsequently, there is less emphasis on parameters that may be difficult to perform in the critically ill or have significant caveats (Valsalva manoeuvres, pulmonary venous Doppler, deceleration time) when compared to the 2009 guidelines.

The presence of diastolic dysfunction in severe sepsis and septic shock has significant clinical implications and hence the importance of a structured diagnostic algorithm as provided by the 2016 ASE/EACVI guidelines. Several studies and a subsequent meta-analysis [4] have indicated an increase in mortality in those patients with diastolic dysfunction, although the current study raises questions about the manner of diagnosis leading to such a conclusion in these studies. One of the many hypotheses surrounding the improved outcomes in the use of beta blockade and noradrenergic sparing agents (i.e. vasopressin) in severe sepsis is that lowering the heart rate may improve diastolic function [23–25]. This may be important as the proposed increased efficiency of diastolic filling in tachycardia (frequency-dependent acceleration of relaxation) is limited in sepsis [26]. One of the largest studies to date highlighted that left ventricular diastolic dysfunction (but not systolic function) had a significant correlation with raised troponins in severe sepsis, which is known to be a predictor of mortality [3]. This relationship of raised troponins and diastolic dysfunction may reflect impaired myocardial relaxation from myocardial oxygen supply demand imbalance, which in turn may be a function of excessive catecholamines, tachycardia or microvascular dysfunction. This potential ischaemia resulting in diastolic dysfunction makes it imperative that myocardial work and oxygen demand are reduced. However, we feel the research to date is significantly impaired due to the lack of a uniform approach to the detection and diagnosis of diastolic dysfunction, which is particularly evident in the meta-analysis by Sanfilippo et al. [4].

It is important for the critical care physician to be able to detect diastolic dysfunction in patients with severe sepsis and septic shock. Despite the relative improved diagnostic capabilities of the 2016 ASE/EACVI guidelines, significant challenges still remain. Firstly, each parameter used in the current guidelines is subject to several caveats. Examples of this include preload dependence [27], the effects of positive pressure ventilation [28] on mitral inflow velocity, and the angle dependence of tissue Doppler [29]. Secondly, several of the parameters are surrogate markers of left atrial pressure, which may not increase acutely in the setting of impaired myocardial relaxation, particularly in sepsis where cardiac dysfunction may exist in the absence of raised filling pressures. For example, there is little known regarding the ability of the left atrium to increase its volume in response to acute changes in pressure due to varying atrial compliance. This is not to discount the value of left atrial volume from the algorithm, as a raised left atrial volume is important if present in differentiating diastolic dysfunction from indeterminate diastolic function. Features of raised left atrial pressure may not be present early in the setting of de novo impaired myocardial relaxation. Herein lies one of the issues when detecting diastolic dysfunction in the critically ill: are we concerned with features of left atrial pressure (which in itself is different to left ventricular end diastolic pressure) which may not be demonstrated early in the patient with de novo diastolic dysfunction due to sepsis, or is the detection of impaired myocardial relaxation (as in *e*′) more important [12]? Furthermore, as recognized by the authors of the current guidelines the cut-off values of parameters used, including that of *e*′, have been validated in patients who are at rest and are not currently under stressed states, as may be seen in the critically ill [16]. Detecting impaired myocardial relaxation in the hyperdynamic circulation is difficult due to the strict cut-off values.

Myocardial relaxation and diastolic function will be abnormal in the setting of systolic dysfunction. This is evident with only 6 and 11% of patients with abnormal systolic function on day 1 and day 3, respectively, having indeterminate diastolic dysfunction (per the 2016 guidelines). Issues may arise, however, when trying to assess the patient with normal systolic function, as noted by the increased proportion of patients with indeterminate diastolic dysfunction in this cohort. Despite the aforementioned limitations, it is the opinion of the authors that future research in this field could use the 2016 ASE/EACVI guidelines as a reference standard for the diagnosis and detection of diastolic dysfunction. By having a consistent framework for the definition of diastolic dysfunction, further research will be strengthened. Such research may revolve around the association with mortality (particularly those with normal systolic function), the impact of fluid balances, ventilation, beta blockade therapy and the comparative use of novel modalities for detecting diastolic dysfunction.

Our study has several limitations. This is a single-centre study and although performed in a unit with an active echocardiographic service, it was not always possible to recruit suitable study patients. A significant proportion of patients with indeterminate diastolic dysfunction based on the 2016 guidelines on day 3 had missing data, which may have changed their grading. Further, a significant proportion of those with normal systolic function had increased myocardial wall thickness, indicating that they would likely have had diastolic dysfunction prior to their ICU presentation. Attempts to clarify pre-existing diastolic dysfunction by searching through patient’s history revealed limited documentation of pre-existing diastolic dysfunction. The authors have not performed a comparison of the two guidelines in ability to predict mortality as firstly, the sample size is too small and secondly the impetus was to focus on how diastolic dysfunction is defined in this cohort, something which is a significant limitation of previous research. Based on our findings, the 2016 ASE/EACVI guidelines could be used in further research to detect if diastolic dysfunction does affect prognosis in severe sepsis and septic shock.

## Conclusion

The 2016 ASE/EACVI guidelines on assessing diastolic function identify a significantly higher incidence of dysfunction in patients with severe sepsis and septic shock compared to the previous 2009 guidelines. Despite limitations, the 2016 ASE/EACVI recommendations appear to have an improved clinical applicability in septic patients relative to the 2009 ASE guidelines. Difficulties remain with recognition of impaired diastolic function in this cohort, particularly those with normal systolic function. Previously published prognostic studies based on diastolic dysfunction in septic patients need to be interpreted with the above findings in mind.

## Additional files

**Additional file 1.** ASE 2009 Guidelines—number of patients with each abnormal parameter.

**Additional file 2.** ASE/EACVI 2016 Guidelines—number of patients with each abnormal parameter.

**Additional file 3.** Severity of systolic dysfunction for patients with abnormal systolic function on day 1 and day 3.

## Abbreviations

*A*: late diastolic velocity of mitral inflow
Ar-a: difference between duration of atrial wave on pulmonary venous Doppler and at mitral annulus
ASE: American Society of Echocardiography
ASE/EACVI: American Society of Echocardiography/European Association of Cardiovascular Imaging
CI: confidence interval
DT: deceleration time
*E*: early diastolic velocity of mitral inflow
*e*′: early diastolic myocardial tissue velocity
*E*/*A*: ratio of early to late diastolic velocity of mitral inflow
*s*′: peak systolic myocardial velocity
*E*/*e*′: ratio of early diastolic mitral inflow velocity to early diastolic myocardial tissue velocity
EF: ejection fraction
LA: left atrium
LV: left ventricle
IQR: interquartile range
PEEP: positive end expiratory pressure
SD: standard deviation
SOFA: sepsis-relating organ failure assessment
TDI: tissue Doppler imaging
TR: tricuspid regurgitation
