# Supplementary material for: Application of updated guidelines on diastolic dysfunction in patients with severe sepsis and septic shock
Source: Ann Intensive Care. 2017 Dec 19;7:121. doi: 10.1186/s13613-017-0342-x (PMC5736511; doi:10.1186/s13613-017-0342-x)
Supplement: Supplementary file 1 — Additional file 1. ASE 2009 Guidelines—number of patients with each abnormal parameter. [file 13613_2017_342_MOESM1_ESM.docx]

**Additional file 1: ASE 2009 Guidelines- number of patients with each abnormal parameter**

|  | **Day 1** | | | | | **Day 3** | | | |  |
| --- | --- | --- | --- | --- | --- | --- | --- | --- | --- | --- |
|  | Normal diastolic function (3) | Grade 1 diastolic dysfunction  (8) | Grade 2 diastolic dysfunction  (0) | Grade 3 diastolic dysfunction  (5) | Indeterminate diastolic function  (46) | Normal diastolic function  (3) | Grade 1 diastolic dysfunction  (7) | Grade 2 diastolic dysfunction  (0) | Grade 3 diastolic dysfunction  (4) | Indeterminate  Diastolic function  (41) |
| Increased LA Volume | 0 | 8 | N/A | 5 | 29 | 1 | 7 | N/A | 4 | 30 |
| Septal e’<8 cm/sec | 0 | 8 | N/A | 5 | 40 | 0 | 7 | N/A | 4 | 31 |
| Lateral e’<10 cm/sec | 0 | 7 | N/A | 3 | 34 | 1 | 5 | N/A | 4 | 32 |
| E/A <0.8 | 2 | 6 | N/A | 0 | 7 | 1 | 2 | N/A | 0 | 8 |
| E/A 0.8-1.5 | 0 | 0 | N/A | 0 | 34 | 2 | 0 | N/A | 0 | 23 |
| E/A >2 | 1 | 0 | N/A | 5 | 4 | 0 | 0 | N/A | 4 | 7 |
| E:e’>13 | 0 | 2 | N/A | 5 | 19 | 0 | 4 | N/A | 3 | 20 |
