# Supplementary material for: Application of updated guidelines on diastolic dysfunction in patients with severe sepsis and septic shock
Source: Ann Intensive Care. 2017 Dec 19;7:121. doi: 10.1186/s13613-017-0342-x (PMC5736511; doi:10.1186/s13613-017-0342-x)
Supplement: Supplementary file 2 — Additional file 2. ASE/EACVI 2016 Guidelines—number of patients with each abnormal parameter. [file 13613_2017_342_MOESM2_ESM.docx]

**Additional file 2: ASE/EACVI 2016 Guidelines- number of patients with each abnormal parameter**

| ­­ | **Day 1** | | | | | **Day 3** | | | |  |
| --- | --- | --- | --- | --- | --- | --- | --- | --- | --- | --- |
|  | Normal diastolic function  (11) | Grade 1 diastolic dysfunction  (14) | Grade 2 diastolic dysfunction  (19) | Grade 3 diastolic dysfunction  (4) | Indeterminate diastolic function  (14) | Normal diastolic function (11) | Grade 1 diastolic dysfunction  (9) | Grade 2 diastolic dysfunction  (22) | Grade 3 diastolic dysfunction  (5) | Indeterminate diastolic function  (7) |
| Increased LA volume | 3 | 8 | 18 | 4 | 9 | 6 | 4 | 22 | 4 | 6 |
| Septal e’<7cm/sec | 2 | 7 | 16 | 4 | 11 | 1 | 5 | 18 | 4 | 5 |
| Lateral e’<10cm/sec | 5 | 9 | 16 | 3 | 11 | 5 | 7 | 21 | 5 | 4 |
| TR velocity > 2.8m/sec | 1 | 1 | 13 | 2 | 0 | 0 | 1 | 13 | 1 | 1 |
| E/A <0.8 | 0 | 6 | 0 | 0 | 1 | 1 | 4 | 4 | 0 | 2 |
| E/A 0.8-2 | 11 | 8 | 16 | 0 | 13 | 7 | 4 | 13 | 0 | 5 |
| E/A >2 | 0 | 0 | 0 | 4 | 0 | 2 | 0 | 0 | 5 | 0 |
| E:e’>14 | 1 | 0 | 8 | 3 | 3 | 0 | 0 | 16 | 4 | 1 |
