# Supplementary figures and images for: Application of updated guidelines on diastolic dysfunction in patients with severe sepsis and septic shock
Source: Ann Intensive Care. 2017 Dec 19;7:121. doi: 10.1186/s13613-017-0342-x (PMC5736511; doi:10.1186/s13613-017-0342-x)

## Slide 1
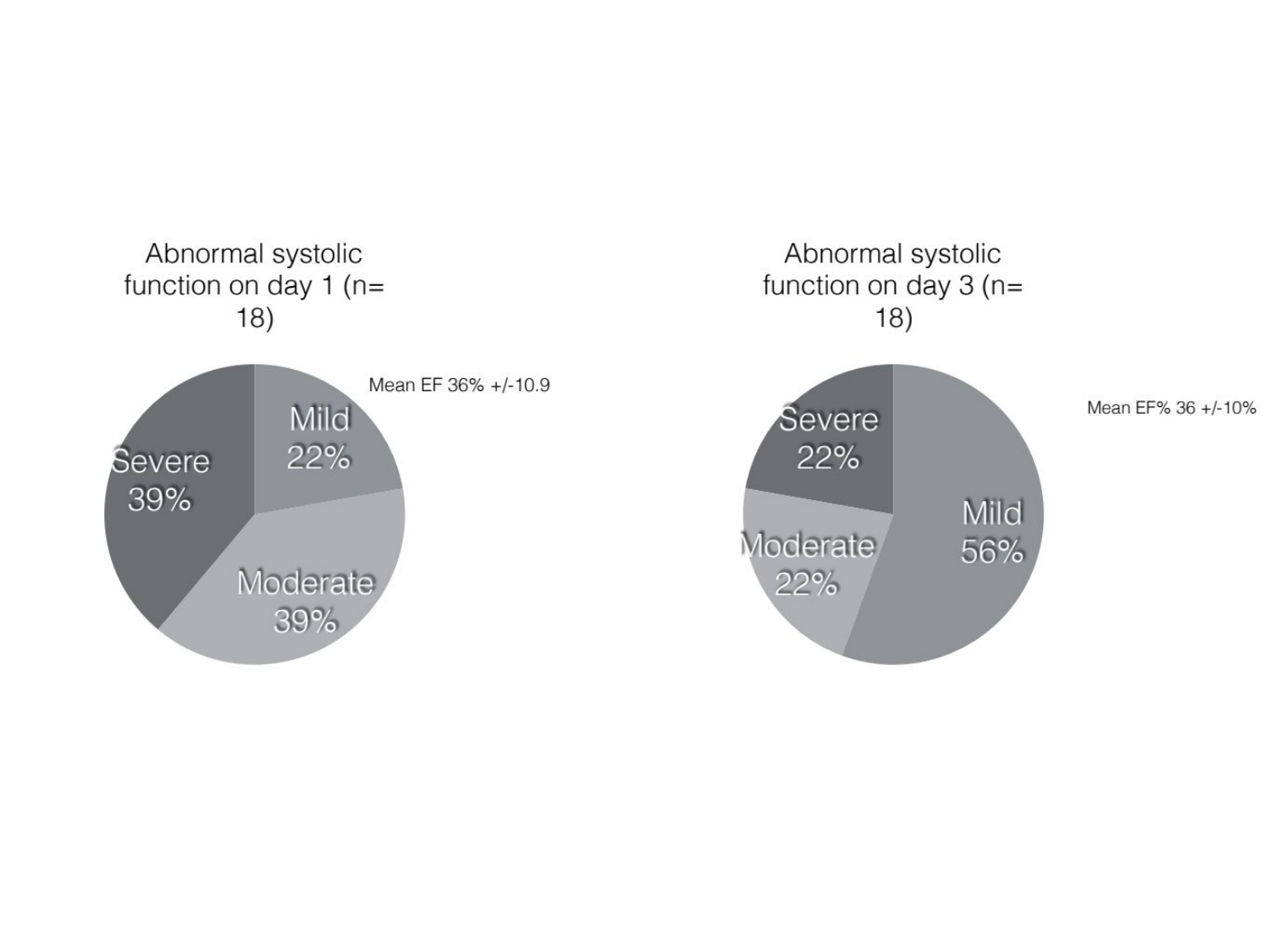

Supplement: Supplementary file 3 — Additional file 3. Severity of systolic dysfunction for patients with abnormal systolic function on day 1 and day 3. [file 13613_2017_342_MOESM3_ESM.pptx]
